# Enhancing Diversity, Equity, and Inclusion in Quantitative Studies of Age and Life Course

**DOI:** 10.1093/geronb/gbad096

**Published:** 2023-09-05

**Authors:** Jessica A Kelley, Roland J Thorpe

**Affiliations:** Case Western Reserve University, Cleveland, Ohio, USA; Johns Hopkins University, Baltimore, Maryland, USA

In 2021, the Editors-in-Chief of six journals of the Gerontological Society of America published an editorial calling for increased attention to diversity, equity, and inclusion (DEI) in the studies of aging and older adults (Meeks et al., 2021). That essay offered resources for authors on inclusive language and labeling, including the Reframing Aging Language guidelines, persons living with dementia, and race/ethnicity. This editorial joined a line of calls for broadening research on aging to expand diversity in study populations, to strive for greater equity in both inquiry and outcomes, and to ensure inclusion through proper language and framing. A few examples include Flatt et al. (2022), who provide recommendations for research on sexual and gender minority older adults. Thorpe and colleagues (2022) lay out steps to overcome structural racism, which they argue is a key barrier that could “affect the scientific enterprise, the conduct and translation of research to benefit diverse populations, and the advancement of scholars of color.” Finally, Baker and Gamaldo (2022) present Best Practices for advancing research among older diverse race and ethnic populations. Yet approaches to addressing diversity, inclusion, and equity in quantitative studies across the life course remain limited.

While efforts at inclusive sampling and measurement are encouraged in studies with primary data collection, the fact is that many scholars in the field of gerontology use secondary data sources, from smaller investigator-generated studies to the large-scale multi-decade panel studies. Investigators utilizing these secondary data sources are often dependent upon the original investigators’ choices of measurement and sample inclusion (and in some cases funder requirements). Thus, even the best-intentioned researchers may still have to list insufficient sample sizes, crude measurement, or low statistical power among their study limitations.

Those challenges acknowledged, the field itself has not always used all available tools that would advance goals of DEI when engaging in secondary data analysis. The purpose of this editorial is to highlight two important barriers to DEI and their consequences when engaging in the study of aging and older adults. We then propose five solutions that could expand diversification and inclusion. To keep our points succinct, we focus our discussion on race and ethnicity in the United States. However, these same principles apply to other aspects of DEI with more global implications, including but not limited to gender and sexual minorities, family types, and persons with disabilities.

## Barrier #1: Erasure of Significant Populations From Age and Life Course Research

The concept of erasure is drawn from cultural and historical paradigms examining social exclusion, defined as the complete absence of voices or groups from discourse (Sasa & Yellow Horse, 2022; Shimkhada et al., 2021). Erasure is used as social critique that certain groups are not just missing, but simply *unseen* in policy, research, or institutional practices.

In the context of research on aging and the life course, certain subgroups of older adults are so regularly excluded from research that their absence does not even raise alarm in the scientific community. Two prime examples in the United States context are Asian Americans and Native/Indigenous persons. In many large-scale cohort studies, these groups have too few observations for meaningful analysis and are so regularly dropped from analysis that the practice is not met with any surprise, much less criticism from reviewers or editors.

A second and emergent issue regarding erasure in studies of aging is a lack of Best Practices standards in accounting for persons who identify as more than one race. Between 2010 and 2020, persons identifying as being of two or more races increased 276% from 9 million to 33.8 million (Jones et al., 2021). Efforts to include mixed-race persons in research have been mired by underrepresentation and ambiguous measurement (see Charmaraman et al., 2014 for extensive discussion). Having a single “multiple race” category or including the racial/ethnic groups with small sample sizes into an “other” race in the analysis, for example, undermines any efforts at meaningful interpretation. A second example is that persons who identify as Hispanic are generally not afforded a racial categorization for the sake of making mutually exclusive race/ethnic categories. Thus, there is no opportunity to explore heterogeneity within Hispanics along the lines of differing ethno-racial identities.

Many of us agree in principle that erasure of certain groups from research on aging is not acceptable. Yet being slow to rectify it allows dangerous—and now largely discounted—assumptions about aging to continue. One such assumption is that aging is a generic human process that proceeds in largely uniform ways across all persons (Dannefer, 1984; Dannefer & Perlmutter, 1990). Acceptance of such implicit assumptions can minimize the urgency to integrate more diverse samples into studies of aging. While research on White, Black, and Hispanic older adults is illustrative and necessary, so is inclusion of groups less seen in research on aging such as Asian Americans, Indigenous persons, and those who identify as more than one race. It is imperative to collect sufficient data across several dimensions of race, ethnic, and nativity to address research questions adequately. These efforts help us to achieve study populations representing the heterogeneity of older adults in the United States.

## Barrier #2: Using Statistical Logic to Explain Social Realities

It is always useful to remind ourselves of the linkage between the development of social statistics and the growing eugenics movement in the late nineteenth and early twentieth centuries (Clayton, 2020). Zuberi and Bonilla-Silva (2008) trace the ways that social statistics were used to reinforce and justify the racial hierarchy of society. By enumerating the deficiencies of persons considered to be not White (a very fluid and unstable definition over time, by the way; Hochschild & Powell, 2008), powerful others could justify discrimination.

Social statistics are a widely accepted tool for understanding social realities today and contribute to our understanding of the diversity of the aging experience. Yet, certain traditions from the origins of statistics are still present today. For example, one oft-used analytic practice is to measure between-group differences by race/ethnic group with the intent on determining whether and by how much the groups differ. Some (Feagin, 2020; Garay & Remedios, 2021) refer to this approach as White-centering, whereby the research question itself implies a White benchmark to which people of color should be compared. In many studies that make race/ethnic comparisons on a select outcome, the arguments often focus on how a particular race or ethnic identity may influence the outcome, all the while leaving Whiteness as a racial or ethnic identity uninterrogated. Further, using White has the default group against which other race/ethnic groups are compared can obscure differences between Hispanic and Black older adults, for example, or assume (incorrectly) that White older adults will always have the best outcomes (Thorpe et al., 2013).

Perhaps even more problematically, the positivist, statistical tradition brings with it a framework grounded in the language of causality (see Holland, 2008 for discussion). In short, to fit complex, intersecting social realities into regression models, investigators often utilize causal language, such as exploring the “effect of race” on a given outcome or whether race/ethnicity moderates a relationship between two other variables (LaVeist, 1994; Zuberi & Bonilla-Silva, 2008). To get some sense of the scope of this problem in scientific discourse, we searched for the phrase “effect of race” in *Google Scholar*. Our search yielded more than 78,000 examples, most of which were in the title of the article. [Thanks to Lilienfeld et al. (2015) for the idea.] Statistically significant differences between groups do not, in themselves, imply causality because their “causal force” may be related to inequality driven by different forms of racism and discrimination (see LaFave et al., 2022a for discussion of how racism affects the health for older African Americans). As a cautionary approach, Lilienfeld et al. (2015) recommend that investigators be explicit that they are “almost always proposing a hypothesis from the data, not drawing a logically justified conclusion from them.”

## Expanding DEI in Studies of Aging

Many in the field of gerontology agree about the urgent need for attention to greater diversity, equality, and inclusion in science (Baker & Gamaldo, 2022; Flatt et al., 2022; Meeks et al., 2021; Thorpe et al., 2022). The great challenge that we face as a field, of course, is accomplishing that goal. Visionary goals such as launching new, large-scale studies with more race/ethnic representation are admirable. However, we should not overlook the value of certain more delimited, strategic goals that can be accomplished quickly, and often with data we have at hand. In what follows we put forth five solutions that have the potential to enhance DEI in quantitative studies of age and the life course.

### Solution #1: Bring on the Variance!

The field of gerontology has seen repeated calls to enhance research on aging and older adults with a stronger focus on variability and heterogeneity (Kelley-Moore & Lin, 2011; Nelson & Dannefer, 1992; Stone et al., 2017), yet inquiry continues, too often, to focus on average differences between groups or average rates of change with age. A first and relatively straightforward step in advancing diversity and inclusion in research on aging is to grow our research questions beyond just studying differences between groups.

If variability increases systematically with age as some researchers have emphasized (Crystal et al., 2017; Dannefer, 2003, 2020; O’Rand, 1996), then to compare age groups on measures of central tendency obscures key features of the aging process. These concerns are not limited to intracohort variability, but extend right down to variability within the individual over time. One recent example incorporating intraindividual variance in trajectories of functional limitations showed that while, at any given age, the average difference in functional limitations between Black and White older adults was less than one-quarter of a unit, the *intraindividual variability* among Black older adults could vary by as many as three units between any two measurement points (Lin & Kelley-Moore, 2017). In other words, Black older adults exhibited less stability in their functional limitations over time than White older adults. Yet this finding would have been missed if we had simply compared average age trajectories between the two groups.

### Solution #2: Focus on Magnitude, Not Significance

Every student in a statistics class learns that the quest of achieving statistical significance is their highest calling. As a result, many studies seek to determine if differences between race/ethnic groups are *significantly* different. However, too many researchers stop short of interpreting the *magnitude* of that difference. McClosky and Ziliak (2010) coined the phrase “cult of statistical significance” to refer to the unfortunate but common practice of conflating statistical significance and substantive difference.

In the context of the tendency to use a White-centered approach, whereby race and ethnic minority older adults are compared to White older adults, the conclusions typically focus on providing statistical proof that persons of color are not White. Without the context of magnitude of differences, a simple *p* < .05 is sufficient to imply race/ethnic groups are worlds apart. Using our example above, Lin and Kelley-Moore (2017) found *statistically significant* differences in average functional limitations between Black and White older adults, but the magnitude was so small that it was uninterpretable. As a field, we should take caution not to overuse words such as “disparity” or “gap” without the proper context of the findings. We recommend Whitfield et al. (2008) for a longer discussion of this issue.

A straightforward solution is for researchers to report—and be fully transparent about—the magnitude of differences between groups prior to launching into a discussion of how race/ethnic groups are different. We also see this as an opportunity for reviewers to pay attention to the tables, figures, and text descriptions of group differences and to challenge authors when the interpretations rely too heavily on the statistical significance rather than the substantive difference.

### Solution #3: Pay Attention to Cohorts

Life course models that integrate indicators from childhood, midlife, and later life are a powerful tool for understanding later-life well-being, but we must take care not to apply these universally, without attention to the sociohistorical and political period in which those life experiences happened. For specific policies or events, the age at which a cohort experiences them can profoundly affect—perhaps even disrupt—life course processes. Failing to incorporate cohort into the problem under study risks over-attributing late-life outcomes to generic (read: cohort invariant) aging processes. Recent work, however, has emphasized the role of cohort membership in explaining patterns in obesity-related morbidities (Kelley & Thorpe, 2021), smoking-related morbidities (Holford et al., 2014), and wealth accumulation (Zewde & Crystal, 2022).

Returning to the question of race and ethnicity in studies of aging, cohort may be an unseen driver of the health and well-being of older racial and ethnic minority older adults. Many older adults who appear in our studies today have had their lives and life chances shaped within the race, ethnic, and immigration milieu operating throughout the twentieth century.

To give just a few examples that affect adults aged 65 and older today: First, older Japanese adults, or their families, may have been subject to U.S. policy to arrest and relocate them to internment camps during from 1942 to 1946 in response to World War II. The profoundly traumatic experience of this racist policy is often integrated into perceptions of successful aging among older Japanese American adults, particularly focused on the need to persevere (Iwamasa & Iwasaki, 2011). Second, The Great Migration in the United States that brought millions of Black Americans to the northern states occurred during the lifetimes of those in our current samples. Research shows that Northbound migrants were positively selected, being younger, healthier, and having more education than those who stayed in the South. These differences between those who stayed and those who migrated translate into differential mortality rates (Black et al., 2015).

Third, many older adults, especially those with roots in Mexico, Philippines, Korea, Cuba, India, and Vietnam, can trace their family immigration story back to the 1965 Immigration and Naturalization Act, which eliminated country-of-origin quotas. Most of these migrants came to the United States either through family unification or occupational immigration, thus having both the social and economic resources to thrive in their new country (Kim & Min, 1992). Understanding differences between pre-1965 and post-1965 immigrants may help to explain observed race/ethnic differences among older adults. One exemplar study is Population-Based Study of Chinese Elderly in Chicago (PINE), which integrates specific questions about immigration into their prospective study (Dong et al., 2014).

### Solution #4: Incorporate Structural Inequality Into Analysis

Federal agencies have launched recent initiatives to address gaps in science on race/ethnic inequality and its impact on health and aging. In 2015, Hill and colleagues (2015) introduced a National Institute on Aging framework for addressing health disparities across the life course through a ­multileveled approach to analysis, including structural causes of inequality. Shortly thereafter, in 2022, NIH launched the UNITE program with the stated goal of dismantling structural and systemic racism (NIH, 2022). One of the themes across these initiatives is the need to move away from merely acknowledging structural racism to measuring and testing it in our analyses. Critics argue that inquiry seems to stall out on the task of documenting differences between race/ethnic groups in outcomes, such as prevalence or incidence of a particular disease, using the logic that any differences not explained by the set of covariates in the model must be due to unobserved structural, societal, or cultural factors. The net effect is to continually reify race/ethnic disparities while the mechanisms remain a black box.

Taking on the task of integrating measures of structural inequality into an analysis is certainly daunting given the complex and integrated forms of structural racism operating across all our social systems (Gee & Hicken, 2021; LaFave et al., 2022b; Szanton et al., 2022). The exercise still has value in expanding our understanding of how racism operates individually and jointly to affect racial/ethnic older adults. Krieger and colleagues demonstrate one potential avenue, in their studies on whether living in the Jim Crow South had adverse effects on the health of Black and White adults (Krieger et al., 2014; Krieger et al., 2017). In another study, researchers incorporate state-level data on racial disparities in illiteracy rates to fully understand the impact of low education on health across race groups (Subramaniam et al., 2009), leading them to conclude that, “Multilevel thinking, grounded in historical and spatiotemporal context, is thus a necessity, not an option” in science on race/ethnicity. Finally, Szanton et al. (2022) discuss the need for developing a comprehensive measure of structural racism that centers the specific experiences of Black Americans rather than developing a universal measure, which may help to better understand the Black–White differences in Alzheimer’s disease and related dementias.

### Solution #5: Integrate New Measurement Modules Into Ongoing Studies

Perhaps more ambitiously, we could expand our data collection and measurement. Ongoing panel studies provide a unique opportunity to gather additional information about racial and ethnic identity and background from respondents. One possibility would be to implement a module based on the Biden administration’s proposed changes to the measurement of race and ethnicity, which would include a separate category for those of Middle Eastern or Northern African descent (Artiga & Pillai, 2023). In the current 1997 United States Office of Management and Budget classification, these persons are categorized as White. Further, detailed race and ethnicity data would allow researchers to begin to develop Best Practices for incorporating persons who identify as more than one race meaningfully into research.

A second data collection possibility would be to implement a life history module on racial/ethnic identity, experiences of discrimination, and colorism. Such research would allow for the lived complexity of people’s ethno-racial identities and potential fluidity of those. Research on adolescents, for example, shows that the context of measurement (filling out forms for school vs describing themselves at home) can lead people to give different answers about their racial and ethnic identity (Hitlin et al., 2006). Formalized “box-checking” exercises, such as during survey interviews, yields substantially less heterogeneity than actually exists in the population. Allowing respondents to “tell the story” of their racial/ethnic identity may help researchers explore heterogeneity among those with the same label or background. Modules could include an extensive battery of questions about race, racial identity, country of origin, and experiences of colorism.

Another potentially fruitful approach is recent development of measures of structural racism at different points across the life course and within different contexts (Dean & Thorpe, 2022; Szanton et al., 2022). For example, LaFave and colleagues (2022b) describe their initial steps in developing a structural racism measure using a life history approach to understand disparities in Alzheimer's Disease and related dementias. This measure underscores the importance of the impact of exposure of structural racism across one’s life across domains. The opportunity to blend qualitative and quantitative approaches to the lived experience of race and ethnicity provides endless possibility.

## Conclusion

The field of gerontology has seen numerous recent calls to expand inquiry about age and the life course to groups that have been historically excluded, overaggregated, or lacked meaningful categorization. Our purpose herein was to focus specifically on those investigators who use secondary data analysis, as this type of research carries unique challenges. When investigators are unable to disaggregate certain race or ethnic categories, or have insufficient observations for meaningful analysis, it may not be immediately clear how one could contribute to the goals diversity or inclusion in science.

To address these barriers, we present five solutions that can be integrated relatively easily into a secondary data analysis. Some may require linking the individual data to structural, historical, or policy indicators. Others may require reframing the research question. Rethinking how to approach studying race and ethnic groups has significant potential to broaden our knowledge base about within-group variability, cohorts and social change, and age patterning in a range of phenomena. Finally, as we note at the outset, these principles and practices apply beyond race and ethnicity, to other forms of social categorization and historic marginalization. Concerted effort toward DEI in studies of aging and older adults requires many hands and creative solutions.
